# Incidence and impact of urogenital sequelae in women following pelvic-ring injuries: a retrospective cohort study

**DOI:** 10.1007/s00264-025-06681-3

**Published:** 2025-11-04

**Authors:** Chin-Chieh Hsu, Chih-Yang Lai, I-Jung Chen, Yung-Heng Hsu, Ying-Chao Chou, Tsia-Shu Lo, Yi-Hsun Yu

**Affiliations:** 1 Department of Obstetrics and Gynecology, Chang Gung Memorial Hospital, Linkou Branch, Chang Gung University, Taoyuan City, Taiwan; 2 Department of Orthopedic Surgery, Musculoskeletal Research Center, Chang Gung Memorial Hospital, Linkou Branch, Chang Gung University, Taoyuan City, Taiwan

**Keywords:** Pelvic ring injury, Urinary tract, Urinary incontinence, Quality of life

## Abstract

**Purpose:**

Pelvic-ring injuries in women often result in urinary dysfunction owing to the proximity of pelvic organs to the urinary tract, significantly affecting quality of life. However, detailed research on urinary sequelae remains limited. This study aimed to assess the incidence of urinary dysfunction in women after pelvic-ring injuries and to identify risk factors influencing urinary function.

**Methods:**

We conducted a retrospective cohort study of women who underwent osteosynthesis for pelvic-ring injuries between January 2022 and June 2023 with ≥ 12 months of follow-up. Urinary dysfunction was evaluated using the Questionnaire for Urinary Incontinence Diagnosis and Female Urinary Symptom Score at one, three, six and 12 months postoperatively.

**Results:**

Fifty-eight patients (mean age, 43.2 years) were included, with motor-vehicle collisions being the most common cause for pelvic-ring injuries (74.1%). Most injuries (84.5%) were classified as Type B. Nearly half of the patients reported urinary symptoms one month post-surgery, which significantly improved over 12 months (*P* < 0.05). In the multivariate analysis, greater injury severity was independently associated with urinary dysfunction at six months (adjusted odds ratio: 1.05, 95% confidence interval 1.00–1.12, *p* = 0.049), while no other clinical or procedural factors, including age, arterial embolisation, or surgical approach, stayed significant. Functional recovery correlated with reduced symptoms over time.

**Conclusion:**

Urinary dysfunction is a frequent but under-recognised complication after pelvic-ring injuries. Although most patients experience gradual improvement over time, greater injury severity is independently associated with early urinary symptoms. Continuous monitoring and timely rehabilitation may help optimise long-term functional recovery.

**Supplementary Information:**

The online version contains supplementary material available at 10.1007/s00264-025-06681-3.

## Introduction

The complexity of injury mechanisms in pelvic ring injuries (PRIs) can lead to intricate fracture patterns, necessitating thorough resuscitation before reconstruction [1–3]. However, associated injuries around the pelvic ring can delay rehabilitation owing to the primary injury and related injury or treatment complications [4–7]. In women, the proximity of pelvic organs to the urinary tract causes a heightened risk for urological complications [4, 7, 8].

The abdominal wall and pelvic floor musculature is integral to maintaining urinary continence by providing structural support to the bladder and urethra and facilitating controlled voiding through coordinated contraction and relaxation [9, 10]. Pelvic-ring symmetry and structural integrity are critical for the optimal function of the pelvic floor and associated urogenital systems [10–12]. PRIs may lead to disruption of pelvic-ring continuity, neural compromise, or vascular injury, which can manifest as urinary dysfunction, including incontinence or retention [4]. Moreover, interventions performed during PRI resuscitation and reconstruction phases may contribute to urinary function impairment [13–15].

The impact on urinary sequelae in women post-PRIs is significant, even without urogenital organ traumatic injuries [4, 8]. Urinary sequelae, including bladder dysfunction, urinary incontinence, and recurrent infections, are common yet under-recognised complications of pelvic-ring fractures. These issues can significantly affect a woman’s quality of life and may require extensive urological management during and after recovery [4, 8, 16]. Despite their frequency and impact, detailed studies on urinary consequences in women post-pelvic trauma remain scarce. This study aimed to determine urinary dysfunction incidence in women post-PRIs and identify potential risk factors for damaged urinary function.

## Materials and methods

### Study design

This is a retrospective cohort analysis of women who underwent osteosynthesis for PRIs at a single medical institute from January 2022 to June 2023. Eligible participants were identified through a review of medical records and included adult women (aged ≥ 18 years) with documented PRIs treated surgically and followed up for at least 12 months. Patients with traumatic urogenital organ injuries, pre-existing urinary conditions, or incomplete follow-up records were excluded.

### Demographic data collection

Data were collected from electronic medical records and images, including demographic information (age and comorbidities), injury characteristics (fracture type and mechanism of PRI, Injury Severity Score [ISS], and New Injury Severity Score [NISS]), types of haemostatic procedure (unilateral or bilateral arterial embolisation [AE]), surgical details (type of osteosynthesis, approach, and any additional procedures), and imaging outcomes.

### Applied PRI classification

Two classification systems were applied for categorising PRIs. The Young and Burgess classification system classifies PRIs based on the direction of the injury force, which leads to specific patterns of pelvic-ring deformity [17]. It includes lateral compression (LC) types I to III, anteroposterior (AP) compression (APC) types I to III, vertical shear (VS), and combined mechanism (CM) injuries. The second classification used for PRIs was the Arbeitsgemeinschaft für Osteosynthesefragen (AO) classification system, which categorises pelvic injuries based on the anatomical and biomechanical characteristics of the fractures [18].

Combining these two classifications for PRIs offers a more comprehensive diagnostic approach by addressing injury mechanisms and fracture anatomy. This dual system aids in treatment planning by tailoring interventions to specific trauma dynamics and structural stability, enabling a more personalised approach. Additionally, it enhances risk assessment by highlighting both force vectors and stability issues, improving outcome predictions.

### Urinary-sequelae assessment

Urinary sequelae were defined as any postoperative urinary dysfunction, including incontinence, urinary retention, and infection. These were assessed during the initial postoperative period and the three month, six month, and one year follow-ups. Data were obtained by adapting the Questionnaire for female Urinary Incontinence Diagnosis (QUID) and the Female Urinary Symptom Score (FUSS) [19, 20].

QUID is a validated, self-administered tool for assessing the type and severity of urinary incontinence in women based on six items that allow distinguishing between stress and urge urinary incontinence according to symptom frequency [19]. FUSS is a brief, self-reported questionnaire for assessing the severity and impact of lower urinary tract symptoms in women based on questions related to symptoms, such as urinary frequency, urgency, incontinence, and nocturia [20].

### Perioperative pelvic-image assessment protocol

Prior to a definite surgical procedure, pelvic imaging—including AP, inlet, and outlet X-ray views and multi-planar computed tomography (mpCT)—was performed to classify fracture types and guide surgical planning. Postoperative imaging was conducted the following day to assess reduction quality by examining fracture gap alignment. The quality of reduction post-osteosynthesis for PRIs was evaluated using the Matta/Tornetta and Levaivre criteria to assess pelvic symmetry in both the vertical and horizontal planes [21, 22]. The Matta/Tornetta criteria quantify reduction quality based on residual vertical displacement on postoperative imaging [21], while the Levaivre criteria evaluate horizontal plane residual deformity [22]. Based on these assessments, the reduction quality between the hemipelvises was categorised as anatomical (difference of 0–5 mm), good (6–10 mm), fair (10–20 mm), or poor (>20 mm).

### Postoperative rehabilitation protocol and functional assessment protocol

Postoperative rehabilitation was conducted according to our institutional protocol (see Supplementary Methods). Functional outcomes were evaluated in all patients at three, six and 12 months post-injury using the Merle d’Aubigné and Majeed scoring systems. The Merle d’Aubigné score assesses pain, mobility, and walking ability, with each domain rated on a scale from 0 (worst) to 6 (best) [23]. The Majeed score, specifically designed for pelvic injuries, includes seven items with a total score ranging from 0 to 100 points [24].

### Statistical analysis

Descriptive statistics were used to summarise the demographic and clinical characteristics of the study population. Continuous variables were reported as median and interquartile range (IQR) or mean and standard deviation (SD), depending on their distribution. Categorical variables were presented as frequencies and percentages. To assess the relationships between continuous variables, Pearson correlation tests were conducted. The Mann–Whitney U test and Kruskal–Wallis test were applied for comparisons between two or more independent groups, respectively.

Univariate and multivariate logistic regression analyses were performed to identify factors associated with urinary dysfunction at six and 12 months, with results expressed as odds ratios (ORs) with 95% confidence intervals (CIs). Linear regression analyses were used to evaluate continuous urinary outcomes (QUID-stress, QUID-urge, and FUSS scores), with results presented as β coefficients and 95% CIs.

For repeated patient-reported outcome (PRO) measures (QUID stress and urge scores and FUSS at 1, 3, 6 and 12 months), a linear mixed-effects model with a random intercept for each patient was used to account for within-subject correlation. Variables with missing 12-month outcomes (Merle d’Aubigné, Majeed, QUID stress and urge, and FUSS scores; five cases each) were handled by complete-case analysis. *P* < 0.05 was considered statistically significant. All analyses were performed using SPSS version 26.0 (IBM, Armonk, NY, USA).

## Results

During the study period, a total of 58 patients were enrolled and analysed. The mean age of the cohort was 43.2 (± 18.7) years. The most frequent cause of PRIs was motor vehicle collisions (74.1%), followed by falls from height (13.8%), pedestrian injuries (5.2%), falls from standing height (5.2%), and crush injuries (1.7%). Extremity fractures were the most common associated injury (39.2%), followed by blunt chest trauma (22.4%) and head injuries (20.7%). Seven patients presented with shock status (systemic blood pressure less than 90 mmHg) during triage. The median ISS was 9 (IQR: 18), while the median NISS was 12.5 (IQR: 18). Emergent AE (emboli material: gelfoam) was performed in eight patients (13.8%) to achieve haemostasis, with six undergoing AE of bilateral internal iliac arteries due to severe hypotension during resuscitation.

The perioperative details of PRIs are summarised in Table 1. According to the Young and Burgess classification system, the LC type was the most prevalent, accounting for 69.0% of cases. Type B PRI under the AO classification system was the most common fracture type (84.5%). Most ventral surgical approaches used the intrapelvic route (78.2%). Open reduction techniques were predominantly used for ventral approaches (87.3%) and closed reduction techniques for dorsal approaches (72.7%). The mean time from injury to surgery was 6.8 ± 9.3 days. The mean duration of Foley catheter placement was 8.5 ± 5.7 days.

**Table 1:** Perioperative details of the 58 enrolled patients

Fracture classification (Young and Burgess)	Number (%)
Lateral compression	40 (69.0)
Anteroposterior compression	9 (15.5)
Vertical shear	9 (15.5)
Fracture classification (AO)	
Type B	49 (84.5)
Type C	9 (15.5)
Surgical approach	
Ventral approach	25 (43.1)
Dorsal approach	3 (5.2)
Combined ventral and dorsal approach	30 (51.7)
Ventral surgical approach	55 (100)
Classified by closed/open reduction	
Closed reduction	6 (10.9)
Open reduction	48 (87.3)
Hybrid	1 (1.8)
Classified by intra-/extrapelvic route	
Intrapelvis	43 (78.2)
Extrapelvis	12 (21.8)
Dorsal surgical approach	33 (100)
Classified by closed/open reduction	
Closed reduction	24 (72.7)
Open reduction	9 (27.3)
Surgery related complications	4 (7.9)

AO: Arbeitsgemeinschaft für Osteosynthesefragen

**Fig. 1:**
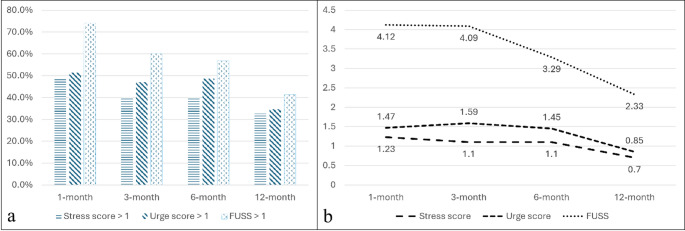
Urinary assessments over time following pelvic-ring injury in women. (**a**) The proportions of patients with stress incontinence (Stress score > 1), urge incontinence (Urge score > 1), and a composite urinary symptom score (FUSS > 1) at the 1-, 3-, 6-, and 12-month follow-up periods. (**b**) The mean scores for stress incontinence, urge incontinence, and FUSS during the same intervals. FUSS: Female Urinary Symptom Score

**Fig. 2:**
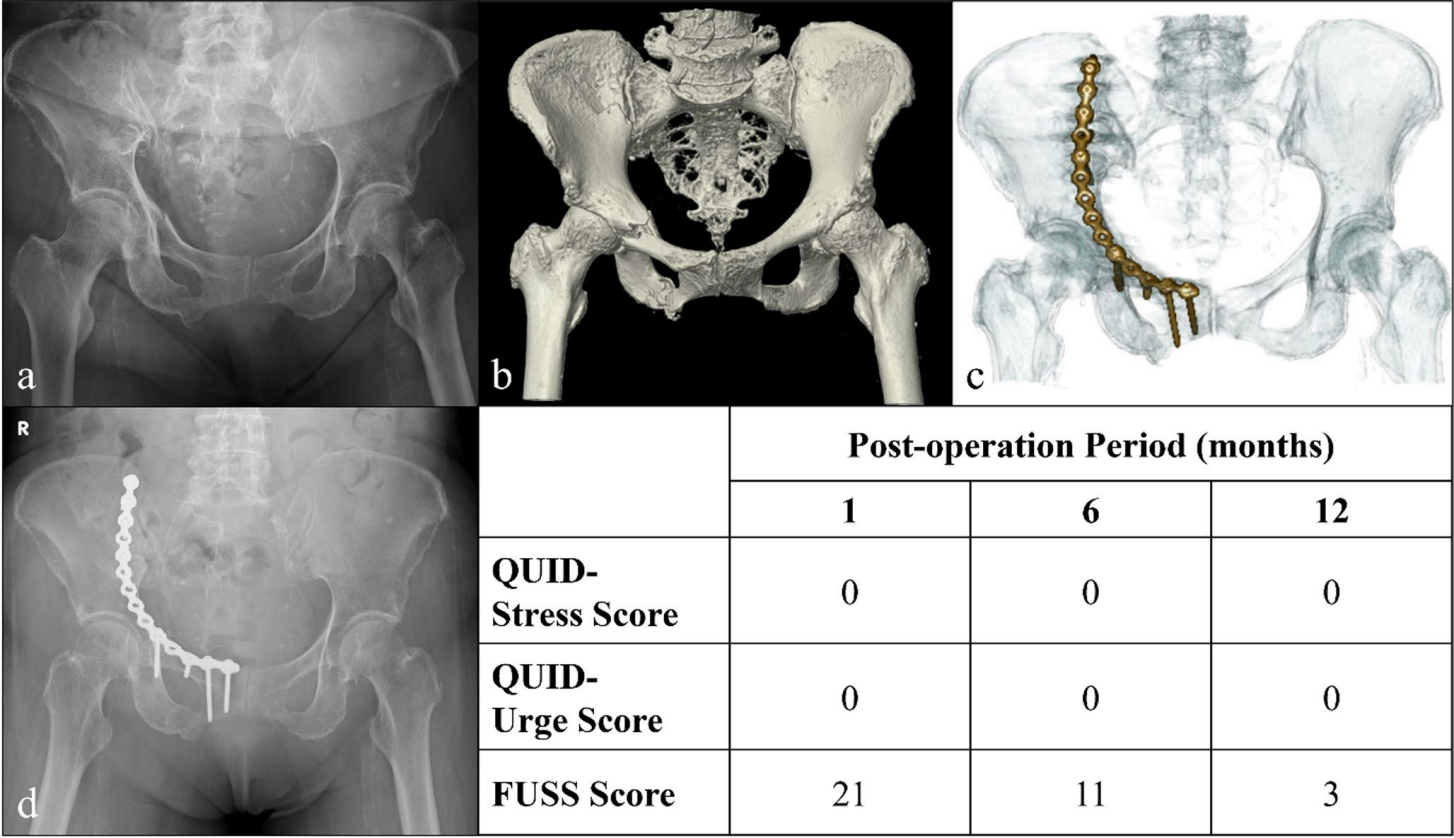
Case presentation of an 84-year-old woman with a PRI classified as LC II (AO B2.2) resulting from a low-energy fall from standing height with related urinary sequelae assessments. (**a**) and (**b**) Preoperative X-ray and CT images. (**c**) Postoperative CT image. (**d**) Follow-up image at 1 year postoperatively

**Fig. 3:**
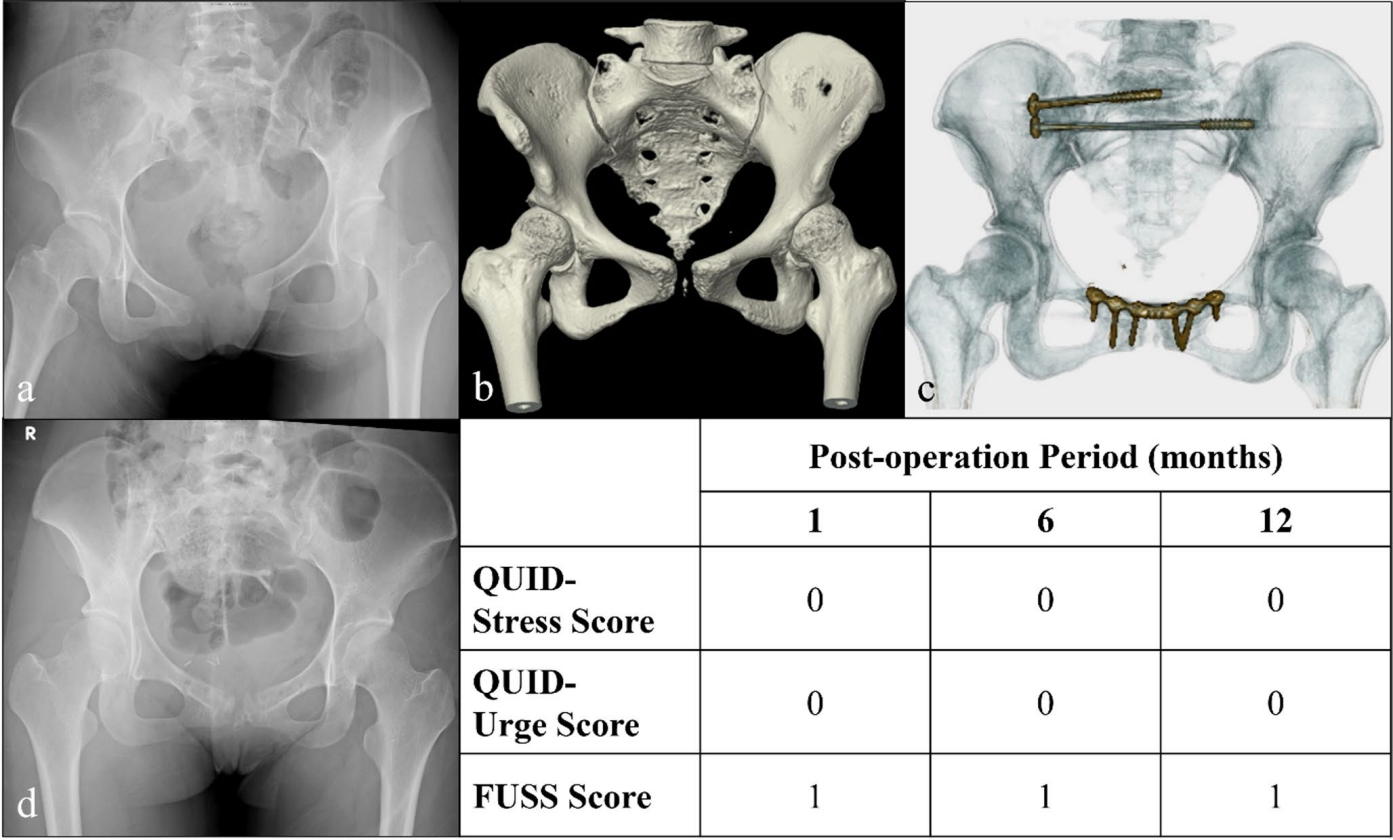
Case presentation of a 21-year-old woman with a PRI classified as APC II (AO B2.3) sustained in a motor vehicle accident with related urinary sequelae assessments. (**a**) and (**b**) Preoperative X-ray (without pelvic binder) and CT scan (with pelvic binder). (**c**) Postoperative CT image. (**d**) One-year postoperative follow-up image

**Fig. 4:**
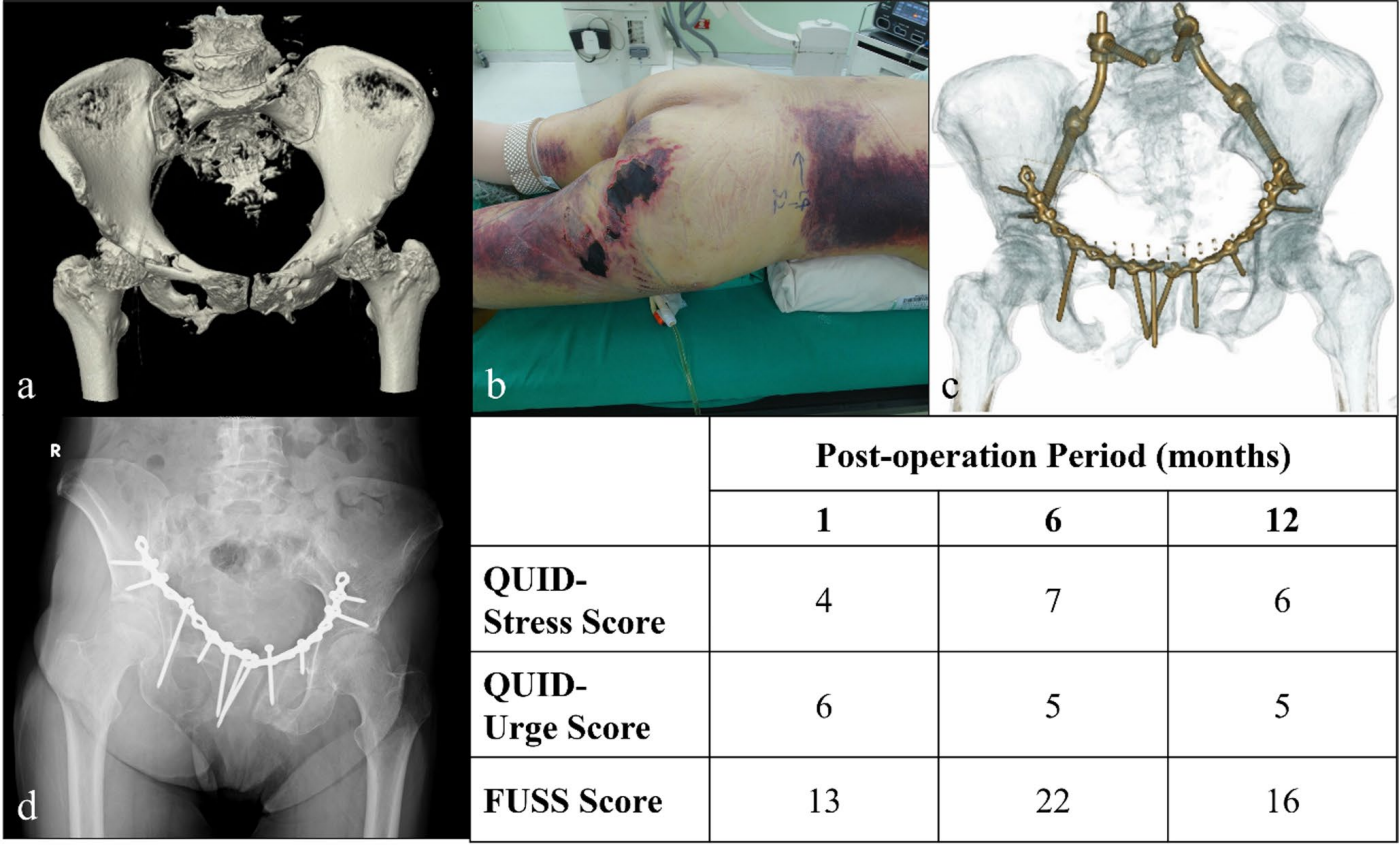
Case presentation of a 62-year-old woman with a VS pelvic ring injury (AO C3.3) sustained in a motor vehicle accident with related urinary sequelae evaluations. (**a**) and (**b**) Preoperative CT scans showing the injury and a Morel-Lavallée lesion involving the right buttock and the posterior thigh. (**c**) Postoperative CT image. (**d**) Follow-up image at 1 year postoperatively

Nearly half of the patients reported residual urinary symptoms 1 month post-injury, as assessed by QUID (stress score: 48.6%; urge score: 51.4%), and 74.1% of patients exhibited symptoms as assessed by FUSS (Fig. 1a). Notably, symptom severity showed significant improvement over time across all evaluation points at one, three, six and 12 months post-injury (Fig. 1b) as evidenced by the repeated measures analysis of variance results (*P* = 0.04 for stress score, 0.02 for urge score and 0.005 for FUSS). Figures 2, 3 and 4 illustrate various fracture patterns along with the analyses of their associated urinary symptoms.

Advanced age, higher ISS and NISS, AE occurrence, ventral approach, intrapelvic approach, and time from injury to surgery were identified as factors contributing to residual urinary symptoms post-osteosynthesis for PRIs (Table 2). Logistic regression analysis revealed that greater injury severity, represented by a higher NISS, was independently associated with urinary symptoms at six months (adjusted OR: 1.05; 95% CI 1.00–1.12; *P* = 0.049), whereas other factors were not significant after multivariate adjustment (Tables 3 and 4). In the linear regression analyses, Foley catheter duration showed a marginal association with higher urinary symptom scores at six months (β = 0.03, 95% CI 0.00–0.06, *P* = 0.08) (Supplementary Tables 1 and 2), suggesting a possible short-term influence of prolonged catheterisation on postoperative urinary function. However, this effect was not sustained at 12 months (β = 0.02, 95% CI − 0.01 to 0.05, *P* = 0.25).

**Table 2:** Correlation analysis of significant factors influencing urinary dysfunction

	QUID questionnaire				FUSS questionnaire		QUID + FUSS	
	Stress score		Urge score					
	*R*	*P*-value	*R*	*P*-value	*R*	*P*-value	*R*	*P*-value
**1-month assessment**								
Age					0.314	0.01	0.28	0.02
Intrapelvic approach		0.036		0.021		0.002		0.002
Injury-to-surgery time (days)	0.299	0.024						
**3-month assessment**								
Age					0.314	0.008	0.285	0.017
ISS					0.351	0.003	0.324	0.006
NISS					0.323	0.006	0.31	0.009
Ventral approach		0.002						
AE						0.006		0.008
Intrapelvic approach				0.003		0.001		0.001
**6-month assessment**								
Age					0.265	0.026	0.249	0.038
AE		0.034		0.03		0.015		0.007
Ventral approach		0.016						
Intrapelvic approach		0.021		0.002		0.001		< 0.001
Injury-to-surgery time (days)	0.28	0.035						
**12-month assessment**								
AE								0.025
Ventral approach		0.003		0.031				0.029
Intrapelvic approach		0.019		0.014		0.002		0.001

AE, arterial embolisation; FUSS, Female Urinary Symptom Score; ISS, Injury Severity Score; NISS, New Injury Severity Score; QUID, Questionnaire for Urinary Incontinence Diagnosis

**Table 3:** Univariate logistic regression analyses for factors associated with urinary dysfunction at 6 and 12 months

Predictors	6-month evaluation				12-month evaluation			
	QUID-Stress	QUID-Urge	FUSS	QUID + FUSS	QUID-Stress	QUID-Urge	FUSS	QUID + FUSS
Age	1.04 (1.00-1.08)^a^, *P* = 0.04*	1.05 (1.01–1.10), *P* = 0.02*	1.05 (1.01–1.10), *P* = 0.02*	1.04 (1.00–1.08), *P* = 0.04*	1.02 (0.97–1.07), *P* = 0.40	1.02 (0.97–1.07), *P* = 0.40	1.04 (0.99–1.09), *P* = 0.10	1.02 (0.97–1.07), *P* = 0.38
NISS	1.06 (1.01–1.12), *P* = 0.03*	1.05 (1.00–1.11), *P* = 0.045*	1.05 (1.00–1.11), *P* = 0.049*	1.05 (1.00–1.12), *P* = 0.05*	1.03 (0.98–1.08), *P* = 0.22	1.03 (0.98–1.08), *P* = 0.21	1.03 (0.98–1.08), *P* = 0.19	1.03 (0.98–1.08), *P* = 0.18
AE	2.4 (1.1–5.8), *P* = 0.03*	2.1 (1.0–4.7), *P* = 0.046*	3.0 (1.2–7.2), *P* = 0.02*	2.5 (1.1–6.0), *P* = 0.04*	1.2 (0.5–3.0), *P* = 0.62	1.3 (0.5–3.0), *P* = 0.58	1.4 (0.5–3.2), *P* = 0.55	1.3 (0.5–3.0), *P* = 0.60
Intrapelvic approach	2.0 (1.0–4.3), *P* = 0.06	1.9 (0.9–3.9), *P* = 0.08	2.8 (1.1–6.9), *P* = 0.04	2.0 (1.0–4.3), *P* = 0.06	1.5 (0.7–3.3), *P* = 0.25	1.5 (0.7–3.2), *P* = 0.24	1.6 (0.7–3.6), *P* = 0.25	1.6 (0.8–3.4), *P* = 0.15
Combined anterior & posterior approach	1.8 (0.9–3.9), *P* = 0.09	1.7 (0.8–3.6), *P* = 0.11	1.8 (0.8–4.2), *P* = 0.12	1.8 (0.9–3.9), *P* = 0.09	1.3 (0.6–2.9), *P* = 0.34	1.4 (0.7–2.9), *P* = 0.28	1.3 (0.6–2.8), *P* = 0.34	1.4 (0.7–2.9), *P* = 0.30
Shock	1.2 (0.6–2.3), *P* = 0.60	1.2 (0.6–2.4), *P* = 0.58	1.3 (0.7–2.4), *P* = 0.54	1.2 (0.6–2.3), *P* = 0.60	1.1 (0.5–2.3), *P* = 0.80	1.1 (0.5–2.3), *P* = 0.80	1.2 (0.5–2.5), *P* = 0.76	1.1 (0.5–2.3), *P* = 0.80
Injury-to-surgery time (days)	1.01 (0.96–1.06), *P* = 0.70	1.01 (0.96–1.06), *P* = 0.68	1.01 (0.97–1.05), *P* = 0.72	1.01 (0.96–1.06), *P* = 0.70	1.00 (0.95–1.05), *P* = 0.95	1.00 (0.95–1.05), *P* = 0.94	1.00 (0.95–1.05), *P* = 0.92	1.00 (0.95–1.05), *P* = 0.95
Foley catheter duration (days)	1.02 (0.99–1.05), *P* = 0.12	1.02 (0.99–1.05), *P* = 0.13	1.02 (0.99–1.05), *P* = 0.10	1.02 (0.99–1.05), *P* = 0.12	1.01 (0.98–1.04), *P* = 0.25	1.01 (0.98–1.04), *P* = 0.26	1.01 (0.98–1.04), *P* = 0.28	1.01 (0.98–1.04), *P* = 0.25
Fracture classification: APC vs. LC	1.4 (0.7–2.9), *P* = 0.31	1.3 (0.6–2.7), *P* = 0.33	1.5 (0.7–3.1), *P* = 0.28	1.4 (0.7–2.8), *P* = 0.29	1.2 (0.6–2.5), *P* = 0.42	1.3 (0.6–2.6), *P* = 0.39	1.4 (0.6–2.9), *P* = 0.37	1.3 (0.6–2.7), *P* = 0.39
Fracture classification: VS vs. LC	1.7 (0.7–3.8), *P* = 0.22	1.5 (0.6–3.6), *P* = 0.26	1.6 (0.7–3.9), *P* = 0.24	1.6 (0.7–3.6), *P* = 0.24	1.4 (0.6–3.2), *P* = 0.30	1.4 (0.6–3.1), *P* = 0.32	1.5 (0.7–3.3), *P* = 0.27	1.4 (0.6–3.1), *P* = 0.31

^a^Statistical results are shown in odds ratio (95% confidence interval), *p* value

*P < 0.05 represents statistical significance

AE, arterial embolisation; APC: anteroposterior compression; FUSS, Female Urinary Symptom Score; LC: lateral compression; NISS: new injury severity score; QUID, Questionnaire for Urinary Incontinence Diagnosis; VS: vertical shear

**Table 4:** Multivariate logistic regression analyses for independent predictors of urinary dysfunction at 6 and 12 months

Predictors	6-month evaluation				12-month evaluation			
	QUID-Stress	QUID-Urge	FUSS	QUID + FUSS	QUID-Stress	QUID-Urge	FUSS	QUID + FUSS
NISS	1.05 (1.00–1.12)^a^, *P* = 0.049*	1.05 (1.00–1.11), *P* = 0.045*	1.05 (1.00–1.11), *P* = 0.049*	1.05 (1.00–1.12), *P* = 0.05	1.03 (0.98–1.08), *P* = 0.22	1.03 (0.98–1.08), *P* = 0.21	1.03 (0.98–1.08), *P* = 0.19	1.03 (0.98–1.08), *P* = 0.18

^a^The statistical results are shown in [odds ratio (95% confidence interval), *p* value]

*P < 0.05 represents statistical significance

FUSS, Female Urinary Symptom Score; NISS: new injury severity score; QUID, Questionnaire for Urinary Incontinence Diagnosis

A linear mixed-effects model was applied to analyse repeated FUSS scores at one, three, six and 12 months, with a random intercept for each patient. Fixed effects included time, age, NISS, arterial embolisation, intrapelvic approach, and combined anterior–posterior approach. The model showed a significant improvement in FUSS scores over time (*P* < 0.001), reflecting progressive recovery of urinary function. Among the covariates, higher NISS was independently associated with worse urinary symptom scores (β=+0.05 per point, 95% CI + 0.01–+0.10, *P* = 0.03), whereas embolisation and surgical approach were not significantly related after adjustment.

When evaluating the quality of pelvic reduction, 52 cases (89.7%) were classified as excellent (*n* = 43) or good (*n* = 9) based on the Matta/Tornetta criteria, while 45 cases (77.9%) were categorised as excellent (*n* = 28) or good (*n* = 17). No significant correlation was found between the quality of reduction and urinary dysfunction (Supplementary Table 3). The association between functional performance and urinary symptoms was analysed (Supplementary Table 4), revealing a consistent negative correlation across all evaluation time points. This finding indicates that a reduction in urinary symptoms over time corresponded with progressive improvement in functional outcomes.

## Discussion

This study evaluated urinary sequelae in a female cohort following surgical treatment for PRIs. Nearly half of 58 participants exhibited urinary symptoms one month post-injury. However, these symptoms showed progressive improvement over time, aligning with functional status enhancements. In the multivariate analysis, higher NISS was the only factor independently associated with early urinary dysfunction at six months, whereas age, embolisation, and surgical approach were not significant factors. The longitudinal mixed-effects model further confirmed significant time-dependent improvement in urinary function across the first postoperative year.

Healthy urinary function correlates with the abdominal wall muscle and pelvic floor physiological presentation. The pelvic floor is a collection of muscles that span the pelvis base, forming a supportive “sling” working alongside surrounding tissues to hold the pelvic organs in their positions [25, 26]. The bladder should not exhibit involuntary contractions during the filling phase, even when provoked [27]. When urinating, the urethral sphincter should relax, allowing the urethra to open with a consistent urine flow. Meanwhile, abdominal wall muscles also generate intraabdominal pressure supporting urinary function [28]. After the insult from PRI and its related management, normal urination physiology may change, resulting in urinary dysfunction.

AE, a haemostatic intervention frequently used during the resuscitative phase of PRI management, has been identified as a potential contributor to urinary dysfunction post-PRIs [29–31]. AE offers notable advantages, including specificity for arterial bleeding, a minimally invasive approach compared to surgical retroperitoneal gauze packing, and a high haemostasis success rate [32, 33]. However, AE is also associated with complications, including urinary dysfunction [14, 34]. Ramasamy et al. [14] reported that up to 50% of patients undergoing AE experienced urinary dysfunction. Nonselective internal iliac artery embolisation has been proposed as a key factor contributing to this sequela [34]. Although AE was not an independent predictor of urinary dysfunction after multivariate adjustment, the observed tendency toward higher symptom scores among embolised patients suggests that ischemic or neural effects may still contribute to transient urinary disturbances. This finding underscores the importance of adopting selective embolisation techniques whenever feasible to preserve pelvic organ perfusion while achieving haemostasis.

In our cohort, a significant proportion of patients (87.3%) underwent ventral approaches for PRI open reduction and internal fixation. Common ventral approaches, including the ilioinguinal, anterior intrapelvic, and pararectus approaches, were used to address ventral and selected dorsal lesions associated with PRIs [35–37]. These techniques involve varying degrees of abdominal wall muscle manipulation—either detachment of the external oblique in the ilioinguinal approach or incision of the rectus abdominis and external oblique aponeurosis in the anterior intrapelvic and pararectus approaches, respectively. Although these procedures may transiently affect lower abdominal or pelvic floor musculature and potentially alter urinary physiology in the immediate postoperative phase, our multivariate analysis did not identify surgical approach as an independent risk factor for urinary dysfunction. This finding suggests that postoperative urinary symptoms are more likely related to overall injury severity and physiological recovery than to surgical corridor.

Osteosynthesis for PRIs primarily aims to achieve symmetric alignment of the bilateral hemipelvises under rigid fixation, thereby preserving pelvic-ring physiological functions, including protection of internal organs and facilitation of weight-bearing ambulation [38]. PRI-related deformities can lead to external compression or elevation of the bladder, potentially reducing its capacity for urine storage and contributing to stress or urgency-related urinary symptoms [4, 39]. In this study, no significant correlation was identified between PRI reduction quality and urinary sequelae. This may be attributed to the consistent application of standard reduction criteria under modern surgical strategy and internal fixator development, which resulted in anatomically restoring pelvic integrity, maintaining structure stability until fracture union, and allowing early weight-bearing ambulation in most cases. Consequently, the potential impact of post-injury pelvic asymmetry on urinary dysfunction may have been mitigated.

These findings emphasise the importance of precise anatomical reduction during osteosynthesis, not only for improving functional mobility in a reasonable time but also for minimising the urinary complication risk in patients recovering from PRIs.

Some limitations must be acknowledged. First, while the functional and urinary questionnaires were prospectively collected, the study was limited by a relatively small sample size and short follow-up duration with missing data. Second, sexual and urinary functions are closely interrelated in women; however, this study did not analyse sexual function as most patients declined to respond, possibly due to cultural factors. Third, decisions on whether to perform AE, its laterality, and the use of gelfoam were made by the emergency physicians based on acute clinical needs; therefore, a formal dose–response analysis was not feasible. Although AE was not an independent predictor in our multivariate analysis, its potential transient influence on pelvic organ perfusion cannot be excluded. Fourth, selection bias may have occurred, with patients experiencing severe injuries or complications being underrepresented. Reliance on patient-reported outcomes also raised the risk of recall bias or underreporting. Finally, although no patient developed a symptomatic urinary tract infection during the index admission, routine urine testing was not performed after discharge; therefore, postoperative urinary tract infections during follow-up cannot be entirely excluded. Although Foley duration showed no lasting effect, transient urinary irritation from prolonged catheterisation cannot be ruled out. Future studies with larger cohorts, longer follow-ups, and culturally sensitive approaches are needed to address these issues.

### Implications for practice and/or policy

Routine assessment using validated instruments such as QUID and FUSS should be integrated into postoperative care. Although surgical or embolisation techniques are not independently associated with dysfunction, greater injury severity warrants closer surveillance and early rehabilitation. Clinicians should emphasise selective embolisation, minimise soft-tissue disruption, and initiate early mobilisation to support recovery. These findings support the development of guidelines for female pelvic trauma management.

## Conclusions

Urinary sequelae may occur in women following pelvic-ring injuries even without direct urinary tract damage. Greater injury severity was the only independent predictor of early urinary dysfunction, while most patients showed gradual recovery over time. Careful surgical technique, selective arterial embolisation, and structured rehabilitation remain essential to optimise pelvic stability, functional recovery, and long-term urinary outcomes.

## Supplementary Information

Below is the link to the electronic supplementary material.

Supplementary Material 1

Supplementary Material 2

Supplementary Material 3

Supplementary Material 4

Supplementary Material 5

## Data Availability

No datasets were generated or analysed during the current study.
