# Supplementary material for: Incidence and impact of urogenital sequelae in women following pelvic-ring injuries: a retrospective cohort study
Source: Int Orthop. 2025 Nov 4;50(1):253–62. doi: 10.1007/s00264-025-06681-3 (PMC12881019; doi:10.1007/s00264-025-06681-3)
Supplement: Supplementary file 1 — Supplementary Material 1 [file 264_2025_6681_MOESM1_ESM.docx]

Supplementary Table 1: Univariate linear regression analyses for continuous urinary function scores at 6 and 12 Months.

|  | 6-Month Evaluation | | | | 12-Month Evaluation | | | |
| --- | --- | --- | --- | --- | --- | --- | --- | --- |
| **Predictors** | **QUID-Stress** | **QUID-Urge** | **FUSS** | **QUID+FUSS** | **QUID-Stress** | **QUID-Urge** | **FUSS** | **QUID+FUSS** |
| Age | β = 0.03 (−0.01 – 0.07),  p = 0.12 | β = 0.04 (0.00 – 0.08),  p = 0.05 | β = 0.05 (0.01 – 0.09),  p = 0.02* | β = 0.04 (0.00 – 0.08),  p = 0.04* | β = 0.02 (−0.02 – 0.06), p = 0.32 | β = 0.02 (−0.02 – 0.06), p = 0.31 | β = 0.03 (−0.01 – 0.07), p = 0.18 | β = 0.02 (−0.02 – 0.06), p = 0.30 |
| NISS | β = 0.06 (0.01 – 0.11), p = 0.02* | β = 0.06 (0.01 – 0.11),  p = 0.03* | β = 0.07 (0.02 – 0.12),  p = 0.01* | β = 0.06 (0.01 – 0.11),  p = 0.02* | β = 0.03 (−0.02 – 0.08), p = 0.26 | β = 0.03 (−0.02 – 0.08), p = 0.24 | β = 0.03 (−0.02 – 0.08), p = 0.24 | β = 0.03 (−0.02 – 0.08), p = 0.23 |
| AE | β = 0.9 (0.2 – 1.6), p = 0.02* | β = 0.8 (0.1 – 1.5), p = 0.03* | β = 1.0 (0.2 – 1.8), p = 0.02* | β = 0.9 (0.2 – 1.6), p = 0.02* | β = 0.3 (−0.4 – 1.0), p = 0.38 | β = 0.3 (−0.4 – 1.0), p = 0.38 | β = 0.4 (−0.3 – 1.1), p = 0.36 | β = 0.3 (−0.4 – 1.0), p = 0.37 |
| Intrapelvic approach | β = 0.6 (−0.1 – 1.3), p = 0.09 | β = 0.7 (0.0 – 1.4), p = 0.06 | β = 0.9 (0.1 – 1.7), p = 0.04* | β = 0.7 (0.0 – 1.4), p = 0.06 | β = 0.4 (−0.3 – 1.1), p = 0.26 | β = 0.4 (−0.3 – 1.1), p = 0.25 | β = 0.5 (−0.2 – 1.2), p = 0.22 | β = 0.4 (−0.3 – 1.1), p = 0.25 |
| Combined anterior & posterior approach | \| β = 0.5 (−0.2 – 1.2), p = 0.15 \| \| --- \| | β = 0.6 (−0.1 – 1.3), p = 0.11 | β = 0.6 (−0.1 – 1.3), p = 0.12 | β = 0.6 (−0.1 – 1.3), p = 0.11 | β = 0.3 (−0.4 – 1.0), p = 0.34 | β = 0.3 (−0.4 – 1.0), p = 0.31 | β = 0.4 (−0.3 – 1.1), p = 0.28 | β = 0.3 (−0.4 – 1.0), p = 0.30 |
| Shock | β = 0.2 (−0.5 – 0.9), p = 0.58 | β = 0.3 (−0.4 – 1.0), p = 0.55 | β = 0.3 (−0.4 – 1.0), p = 0.53 | β = 0.3 (−0.4 – 1.0), p = 0.56 | β = 0.1 (−0.6 – 0.8), p = 0.80 | β = 0.1 (−0.6 – 0.8), p = 0.80 | β = 0.2 (−0.5 – 0.9), p = 0.78 | β = 0.1 (−0.6 – 0.8), p = 0.80 |
| Injury to surgery (day) | β = 0.01 (−0.04 – 0.06), p = 0.74 | β = 0.00 (−0.05 – 0.05), p = 0.95 | β = 0.01 (−0.04 – 0.06), p = 0.70 | β = 0.01 (−0.04 – 0.06), p = 0.72 | β = 0.00 (−0.05 – 0.05), p = 0.93 | β = 0.00 (−0.05 – 0.05), p = 0.94 | β = 0.01 (−0.04 – 0.06), p = 0.91 | β = 0.00 (−0.05 – 0.05), p = 0.93 |
| Foley catheter duration (day) | β = 0.03 (−0.00 – 0.06), p = 0.08 | β = 0.03 (−0.00 – 0.06), p = 0.09 | β = 0.03 (−0.00 – 0.06), p = 0.09 | β = 0.03 (−0.00 – 0.06), p = 0.08 | β = 0.02 (−0.01 – 0.05), p = 0.25 | β = 0.02 (−0.01 – 0.05), p = 0.26 | β = 0.02 (−0.01 – 0.05), p = 0.25 | β = 0.02 (−0.01 – 0.05), p = 0.25 |
| Fracture classification: APC vs. LC | β = 0.4 (−0.3 – 1.1), p = 0.26 | β = 0.4 (−0.3 – 1.1), p = 0.27 | β = 0.5 (−0.2 – 1.2), p = 0.23 | β = 0.4 (−0.3 – 1.1), p = 0.25 | β = 0.3 (−0.4 – 1.0), p = 0.39 | β = 0.3 (−0.4 – 1.0), p = 0.38 | β = 0.3 (−0.4 – 1.0), p = 0.36 | β = 0.3 (−0.4 – 1.0), p = 0.38 |
| Fracture classification: VS vs. LC | β = 0.5 (−0.3 – 1.3), p = 0.24 | β = 0.5 (−0.3 – 1.3), p = 0.24 | β = 0.6 (−0.2 – 1.4), p = 0.22 | β = 0.5 (−0.3 – 1.3), p = 0.23 | β = 0.4 (−0.4 – 1.2), p = 0.31 | β = 0.4 (−0.4 – 1.2), p = 0.30 | β = 0.5 (−0.3 – 1.3), p = 0.28 | β = 0.4 (−0.4 – 1.2), p = 0.30 |

*p < 0.05 represents statistical significance

NISS: new injury severity score; AE: arterioembolization; APC: anteroposterior compression; LC: lateral compression; VS: vertical shear
