# Supplementary material for: Incidence and impact of urogenital sequelae in women following pelvic-ring injuries: a retrospective cohort study
Source: Int Orthop. 2025 Nov 4;50(1):253–62. doi: 10.1007/s00264-025-06681-3 (PMC12881019; doi:10.1007/s00264-025-06681-3)
Supplement: Supplementary file 2 — Supplementary Material 2 [file 264_2025_6681_MOESM2_ESM.docx]

Supplementary Table : Multivariate linear regression analyses for continuous urinary function scores at 6 and 12 Months.

|  | 6-Month Evaluation | | | | 12-Month Evaluation | | | |
| --- | --- | --- | --- | --- | --- | --- | --- | --- |
| **Predictors** | **QUID-Stress** | **QUID-Urge** | **FUSS** | **QUID+FUSS** | **QUID-Stress** | **QUID-Urge** | **FUSS** | **QUID+FUSS** |
| NISS | β = 0.06 (0.01 – 0.11), p = 0.03* | β = 0.06 (0.01 – 0.11), p = 0.04* | β = 0.07 (0.02 – 0.12), p = 0.02* | β = 0.06 (0.01 – 0.11), p = 0.03* | β = 0.03 (−0.02 – 0.08), p = 0.26 | β = 0.03 (−0.02 – 0.08), p = 0.24 | β = 0.03 (−0.02 – 0.08), p = 0.24 | β = 0.03 (−0.02 – 0.08), p = 0.23 |
| AE | β = 0.7 (−0.1 – 1.5), p = 0.08 | β = 0.6 (−0.2 – 1.4), p = 0.09 | β = 0.8 (−0.0 – 1.6), p = 0.06 | β = 0.7 (−0.1 – 1.5), p = 0.08 | β = 0.3 (−0.4 – 1.0), p = 0.36 | β = 0.3 (−0.4 – 1.0), p = 0.35 | β = 0.4 (−0.3 – 1.1), p = 0.32 | β = 0.3 (−0.4 – 1.0), p = 0.34 |
| Foley catheter duration (day) | β = 0.03 (−0.00 – 0.06), p = 0.08 | β = 0.03 (−0.00 – 0.06), p = 0.09 | β = 0.03 (−0.00 – 0.06), p = 0.09 | β = 0.03 (−0.00 – 0.06), p = 0.08 | β = 0.02 (−0.01 – 0.05), p = 0.25 | β = 0.02 (−0.01 – 0.05), p = 0.26 | β = 0.02 (−0.01 – 0.05), p = 0.25 | β = 0.02 (−0.01 – 0.05), p = 0.25 |

*p < 0.05 represents statistical significance

NISS: new injury severity score; AE: arterioembolization.
