# Supplementary material for: Incidence and impact of urogenital sequelae in women following pelvic-ring injuries: a retrospective cohort study
Source: Int Orthop. 2025 Nov 4;50(1):253–62. doi: 10.1007/s00264-025-06681-3 (PMC12881019; doi:10.1007/s00264-025-06681-3)
Supplement: Supplementary file 3 — Supplementary Material 3 [file 264_2025_6681_MOESM3_ESM.docx]

Supplementary Table 3. Correlation analysis between the reduction quality of the pelvic ring and urinary assessment.

|  | **QUID questionnaire (P-value)** | | **FUSS questionnaire (P-value)** | **QUID + FUSS (P-value)** |  |
| --- | --- | --- | --- | --- | --- |
|  | **Stress score** | **Urge score** |  |  |  |
| **1-month assessment** | | | | | |
| **Matta/Tornetta criteria** | 0.26 | 0.23 | 0.48 | 0.30 |  |
| **Lefaivre criteria** | 0.94 | 0.72 | 0.97 | 0.90 |  |
| **3-month assessment** | | | | | |
| **Matta/Tornetta criteria** | 0.24 | 0.16 | 0.14 | 0.10 |  |
| **Lefaivre criteria** | 0.26 | 0.46 | 0.50 | 0.38 |  |
| **6-month assessment** | | | | | |
| **Matta/Tornetta criteria** | 0.93 | 0.55 | 0.98 | 0.92 |  |
| **Lefaivre criteria** | 0.90 | 0.68 | 0.53 | 0.64 |  |
| **12-month assessment** | | | | | |
| **Matta/Tornetta criteria** | 0.48 | 0.45 | 0.34 | 0.33 |  |
| **Lefaivre criteria** | 0.97 | 0.49 | 0.21 | 0.33 |  |

FUSS, Female Urinary Symptom Score; QUID, Questionnaire for Urinary Incontinence Diagnosis
