# Supplementary material for: Incidence and impact of urogenital sequelae in women following pelvic-ring injuries: a retrospective cohort study
Source: Int Orthop. 2025 Nov 4;50(1):253–62. doi: 10.1007/s00264-025-06681-3 (PMC12881019; doi:10.1007/s00264-025-06681-3)
Supplement: Supplementary file 4 — Supplementary Material 4 [file 264_2025_6681_MOESM4_ESM.docx]

Supplementary Table 4. Correlation analysis between the functional outcome assessment and urinary assessment.

|  |  | **Merle d'Aubigné score** | | | **Majeed score** | | |
| --- | --- | --- | --- | --- | --- | --- | --- |
| **Months** | | **3** | **6** | **12** | **3** | **6** | **12** |
| **QUID**  **3 months** | **Stress score** | R = -0.334  P = 0.005 |  |  | R = -0.371  P = 0.002 |  |  |
|  | **Urge score** | - |  |  | - |  |  |
| **QUID**  **6 months** | **Stress score** |  | R = -0.293  P = 0.014 |  |  |  |  |
|  | **Urge score** |  | R = -0.301  P = 0.011 |  |  |  |  |
| **QUID**  **12 months** | **Stress score** |  |  | - |  |  | - |
|  | **Urge score** |  |  | R = -0.359  P = 0.011 |  |  | - |
| **FUSS**  **3 months** | | R = -3.86  P = 0.001 |  |  | R = -0.468  P < 0.001 |  |  |
| **`FUSS**  **6 months** | |  | R = -0.387  P = 0.001 |  |  | R = -0.347  P = 0.003 |  |
| **FUSS**  **12 months** | |  |  | R = -0.297  P = 0.038 |  |  | - |
| **QUID + FUSS**  **3 months** | | R = -0.387  P = 0.001 |  |  | R = -0.471  P < 0.001 |  |  |
| **QUID + FUSS**  **6 months** | |  | R = -0.377  P = 0.001 |  |  | R = -0.319  P = 0.007 |  |
| **QUID + FUSS**  **12 months** | |  |  | R = -0.339  P = 0.017 |  |  | - |

FUSS, Female Urinary Symptom Score; QUID, Questionnaire for Female Urinary Incontinence Diagnosis
