# Supplementary material for: Incidence and impact of urogenital sequelae in women following pelvic-ring injuries: a retrospective cohort study
Source: Int Orthop. 2025 Nov 4;50(1):253–62. doi: 10.1007/s00264-025-06681-3 (PMC12881019; doi:10.1007/s00264-025-06681-3)
Supplement: Supplementary file 5 — Supplementary Material 5 [file 264_2025_6681_MOESM5_ESM.docx]

***Postoperative rehabilitation protocol***

Postoperative management post-osteosynthesis for PRIs is critical for optimising recovery, minimising complications, and enhancing functional outcomes. Early mobilisation is a cornerstone of postoperative care to reduce joint stiffness and muscle atrophy. Rehabilitation is initiated within the first postoperative day, beginning with non-weight-bearing or partial weight-bearing exercises, dependent on the stability of the fixation and clinical tolerance. Sometimes, ambulation with a wheelchair is necessary for patients with concomitant injuries.

A Foley catheter was routinely placed in patients suspected of or diagnosed with PRI upon presentation to the emergency department. The Foley catheter remained in situ throughout the surgical procedure to enhance operational safety, maintain intraoperative bladder decompression, and monitor urine amount. Post-surgery, the catheter is typically removed 2 days postoperatively, contingent upon the absence of any contraindications, such as urinary tract infections or other complications that may require prolonged catheterisation.

Pharmacological prophylaxis for venous thromboembolism (VTE) and heterotopic ossification (HO) was not routinely administered. Owing to the relatively low incidence of VTE post-PRI in our population [40], mechanical prophylaxis using compression stockings was employed for 12 weeks. Patients with positive findings on routine postoperative lower-extremity venous ultrasonography were treated with low-molecular-weight heparin, followed by oral anticoagulants after discharge. For HO management, oral indomethacin was prescribed only upon radiographic evidence of HO development.
